# Electrocardiographic Screening of Arrhythmogenic Cardiomyopathy in Genotype-Positive and Phenotype-Negative Relatives

**DOI:** 10.3389/fcvm.2021.646391

**Published:** 2021-05-07

**Authors:** Jose Maria Lopez-Ayala, Javier Gimeno-Blanes, David Lopez-Cuenca, Maria Sabater Molina, Juan Ramon Gimeno-Blanes

**Affiliations:** ^1^Department of Cardiology, San Juan General University Hospital, Alicante, Spain; ^2^Department of Signal Theory and Communications, Miguel Hernández University, Alicante, Spain; ^3^Department of Cardiology, Virgen de la Arrixaca University Hospital, Murcia, Spain

**Keywords:** arrhythmogenic right ventricular cardiomyopathy, electrocardiogram, genetic carrier, early diagnosis, familiar screening

## Abstract

**Background:** Arrhythmogenic cardiomyopathy is a hereditary cause of ventricular arrhythmias and sudden death. Identifying the healthy genetic carriers who will develop the disease remains a challenge. A novel approach to the analysis of the digital electrocardiograms of mutation carriers through signal processing may identify early electrocardiographic abnormalities.

**Methods:** A retrospective case–control study included a population of healthy genetics carriers and their wild-type relatives. Genotype-positive/phenotype-negative individuals bore mutations associated with the development of arrhythmogenic cardiomyopathy. The relatives included had a non-pathological 12-lead electrocardiogram, echocardiogram, and a cardiac magnetic resonance. Automatic digital electrocardiographic analyses comprised QRS and terminal activation delay duration, the number of QRS fragmentations, ST slope, and T-wave voltage.

**Results:** Digital 12-lead electrocardiograms from 41 genotype-positive/ phenotype-negative (29 simple carriers and 12 double mutation carriers) and 73 wild-type relatives were analyzed. No differences in the QRS length, the number of QRS fragmentations, and the voltage of the T-wave were observed. After adjusting for potential confounders, double carriers showed an average ST-slope flatter than those of the simple carriers and wild type [5.18° (0.73–8.01), 7.15° (5.14–11.05), and 11.46° (3.94–17.49), respectively, *p* = 0.005]. There was a significant negative correlation between the ST slope and the age in genotype-positive/phenotype-negative relatives (*r* = 0.376, *p* = 0.021) not observed in their wild-type counterparts (*r* = 0.074, *p* = 0.570).

**Conclusions:** A flattened ST segment may be an early sign of electrical remodeling that precedes T-wave inversion in healthy genetic carriers. A thorough analysis of the digital electrocardiographic signal may help identify and measure early electrical abnormalities.

## Introduction

Arrhythmogenic cardiomyopathy is a cause of unexpected sudden death and ventricular arrhythmias in the general population including the youth and competitive athletes (1, 2). This umbrella term encompasses a broad phenotypic spectrum of disease that may predominantly affect one of the ventricles or both (3). The underlying cause of the disease is usually a mutation in a desmosomal gene, although mutations in other genes have been also described (4). Most common forms of arrhythmogenic cardiomyopathy are classically inherited in a dominant trait with incomplete penetrance and a variable clinical phenotype. The natural history of the disease encompasses a concealed, an electric, and a late structural phase, being sinister arrhythmias and sudden death a potential outcome at any stage even in the absence of overt structural abnormalities (5).

Once the diagnosis of arrhythmogenic cardiomyopathy is established, carrying out a complete family screening is recommended. As electrocardiographic changes usually precede the development of subsequent structural abnormalities, it is paramount to identify early electrocardiographic abnormalities that herald an initial electric phase of the disease. Thus far, predicting the individuals who will develop a severe phenotype remains a challenge.

As desmosomal disruption results in cell-to-cell mechanical and electrical uncoupling and fibrofatty replacement that slow down the electrical propagation (6), we hypothesized that genotype-positive/phenotype-negative relatives may show longer QRS terminal activation delays and minor ST/T-wave abnormalities preceding the development of the overt electrocardiographic abnormalities included in the current diagnostic criteria (7). The goal of this research is, therefore, to identify early electrocardiographic abnormalities in genotype-positive/phenotype-negative relatives.

## Materials and Methods

We carried out a retrospective electrocardiographic evaluation in genotype-positive/phenotype-negative relatives of families with a previous diagnosis of arrhythmogenic cardiomyopathy. All individuals had been previously assessed in a monographic cardiomyopathy clinic, were above 18 years of age, and consented to undergo genetic testing. None of the included individuals reported a previous history of heart disease. Participants showed normal imaging findings (non-dilated left and right ventricles, normal ejection fraction, no regional wall motion abnormalities, and no late gadolinium enhancement on cardiac magnetic resonance, **Table 2**). Mutation carriers and controls showing inverted T-waves in precordial leads beyond V1 were excluded from the analysis.

### Genetic Testing

A panel of genes associated with arrhythmogenic cardiomyopathy was tested in the proband of each family (defined as the first member to be diagnosed with the disease). Genetic confirmation tests were performed through Sanger sequencing in all the relatives. The frequency of the mutations in the general population was evaluated with the Genome Aggregation Database (gnomAD) (RRID:SCR_014964) Frequencies < 0.001 supports the pathogenicity of the included variants. American College of Medical Genetics Score (ACMG) score and familiar cosegregation were also used to establish the clinical significance of the mutations.

### Digital Electrocardiographic Analysis

Digital 12-lead electrocardiograms were obtained with a GE MAC 5000 System. Fiducial points were independently set for each beat in the 12 leads with MATLAB 2010 [Version 7.10.0 (R2010a), Natick, Massachusetts: The MathWorks Inc, MATLAB, RRID: SCR_001622]. The cardiology analyst (JL) was blinded to the genetic data at the time of evaluation. Details about signal processing have been published elsewhere (8). In summary, fiducial points were set at the beginning of the P-wave, QRS onset, QRS offset, and at the end of the T-wave. The terminal activation delay of the QRS was measured from the nadir of the S-wave to the J-point. The T-wave voltage (mV) and area (μV·s) were calculated through changes in the slope of the electrocardiographic signal. The ST slope was defined as the averaged angle between the ST tangent with the baseline in the 12 leads. The investigated electrocardiographic variables were the median QRS duration, terminal activation delay in V1–V3, the number of QRS fragmentations, the presence of bundle branch blocks, ST-slope, and T-wave voltage and area.

### Statistical Analysis

Qualitative variables were expressed in percentage and compared with Pearson's χ^2^-test (or Fisher's test when appropriate). Quantitative variables are described as mean ± standard deviation (or median and interquartile rank should they do not follow a normal distribution), and they were compared with the Student's *t*-test or ANOVA (or the non-parametric Wilcoxon's and Kruskal–Wallis tests). Normality was tested with the Shapiro–Wilk test. The correlation between two quantitative variables was determined with Pearson's *r* coefficient.

The effect of genetic status (wild type, a single mutation, or double mutation) on the ST slope and the effect of potential confounding variables (age, sex, body mass index, predicted effect of the mutation) was determined with a linear regression model. Wild-type status and female sex were set as reference categories. Statistical analysis was performed with the package Stata 13.1 (Copyright 1985-2013 Stata Corp Texas 77845 USA, RRID: SCR_012763).

## Results

### Genetic Results

A total of 41 genotype-positive/phenotype-negative relatives with a digital electrocardiogram were included in the study. Pathogenic mutations were identified in five desmosomal genes (*DSP, PKP-2, DSG-2, DSC-2, JUP*) and *LDB3*. The frequency and the proposed mechanism of each mutation are described in Table 1.

**Table 1:** List of the 41 identified mutations in the desmosomal genes and *LDB3*.

**Gene**	**Mutation**	***N***	**Mechanism**	**Affected carriers**	**MAF in gnomAD (genome)**	**ACMG score**
*JUP*	c.56C>T (p.Thr19Ile)	3	Missense	3	3.234E−5	Uncertain significance
*PKP-2*	c.419C>T (p.Ser140Phe)	2	Missense	7	0.0019	Uncertain significance
	c.1253C>A (p.Ala418Asp)	1	Missense	1	–	Uncertain significance
*DSC-2*	c.2686_2687dup (p.Ala897Lysfs*900)	4	Nonsense/truncation	5	–	Pathogenic
	c.397G>A (p.Ala133Thr)	1	Missense	1	6.459E−5	Uncertain significance
	c.1350A>G (p.Arg450=)	1	Missense	2	0.0019	Likely benign
*DSP*	c.5773C>T (p.Gln1925*)	2	Nonsense/truncation	1	–	Pathogenic
	c.3219_3220insA (p.Ala1074Serfs*1087)	5	Nonsense/truncation	5	–	Pathogenic
	c.2631-1G>A (IVS18-1G>A)	1	Splicing/intronic	1	–	Pathogenic
	c.1339C>T (p.Q447X)	4	Nonsense/truncation	20	–	Pathogenic
	c.4609C>T (p.Arg1537Cys)	7	Missense	6	0.0087	Benign
	c.4372C>G (p.Arg1458Gly)	3	Missense	3	0.0009	Uncertain significance
*DSG-2*	c.2759T>G (p.Val920Gly)	3	Missense	7	0.0033	Benign
	c.166G>A (p.Val56Met)	2	Missense	1	0.0016	Likely benign
*LDB3*	c.1051A>G (p.Thr351Ala)	2	Missense	6	0.0003	Likely benign

*Nucleotide and the protein changes are shown along with the predicted effect of the mutation. The mechanism of the mutation is classified as missense, nonsense, or truncation and boundary mutations affecting the splicing. Affected carriers represents the total number of probands and relatives bearing the mutation. Minor allele frequency (MAF) in the gnomAD database.*

*JUP, junctional plakoglobin; PKP-2, plakophilin-2; DSC-2, desmocollin-2; DSP, desmoplakin; DSG-2, desmogleín-2; LDB3, LIM domain binding 3; ACMG score, American College of Medical Genetics Score*.

### Electrocardiographic Characterization in Mutation Carriers

Basal characteristics and electrocardiographic findings are summarized in Table 2. No significant differences were observed in the median QRS length, QRS terminal activation delay, or the number of fragmentations in the QRS between genotype-positive/phenotype-negative and wild-type relatives. Despite the ST slope being flatter in mutation carriers compared to wild type, this difference was not significant, 6.24° (4.11–9.81) vs. 11.94° (6.19–17.53), *p* = 0.187. No differences were observed either in the T-wave voltage, T-wave area, or T-wave dispersion between groups.

**Table 2:** Quantitative analysis of the electrocardiographic parameters through digital analysis in gene carriers and wild-type relatives.

	**Mutation carriers (*N* = 41)**	**Wild type (*N* = 73)**	***p***
Sex (male)	22 (53.7%)	45 (61.64%)	0.939
Age (years)	38.5 (25–50.5)	34 (26–47)	0.514
BMI (kg/m^2^)	23.67 (22.66–25.50)	23.41 (21.80–26.85)	0.880
LVED volume (ml)	137 ± 19	132 ± 9	0.854
LVEF (%)	63 ± 8	61 ± 5	0.756
RVED volume (ml)	132 ± 9	134 ± 6	0.698
RVEF (%)	60 ± 6	59 ± 7	0.921
**ECG**			
IRBBB	14 (34.15%)	23 (31.51%)	0.977
CRBBB	3 (6.82%)	3 (4.11%)	0.166
QRS (ms)	84 (78–90)	84 (80–84)	0.803
QRS V1–3 (S-offset) (ms)	38 (32–44)	38 (34–42)	0.474
QRS fragmentation (*n*)	1 (0–2)	1 (0–1)	0.268
ST slope (°)	6.24 (4.11–9.81)	11.46 (3.94–17.49)	0.187
TWI (*n*)	1 (1, 2)	1 (1, 2)	0.896
T-wave amplitude (mV)	0.20 (0.11–0.30)	0.214 (0.139–0.285)	0.348
T-wave area (μV·s)	18.58 (9.13–30.42)	22.35 (12.77–32.98)	0.478
T-wave dispersion (μV·s)	2.50 (1.48–5.02)	1.82 (0.81–4.79)	0.742

*Values are expressed in percentage, median, and interquartile range.*

*IRBBB, incomplete right bundle branch block; CRBBB, complete right bundle branch block; TWI, T-wave inversion*.

### Electrocardiographic Findings in Double-Mutation Carriers

**Figure 1:**
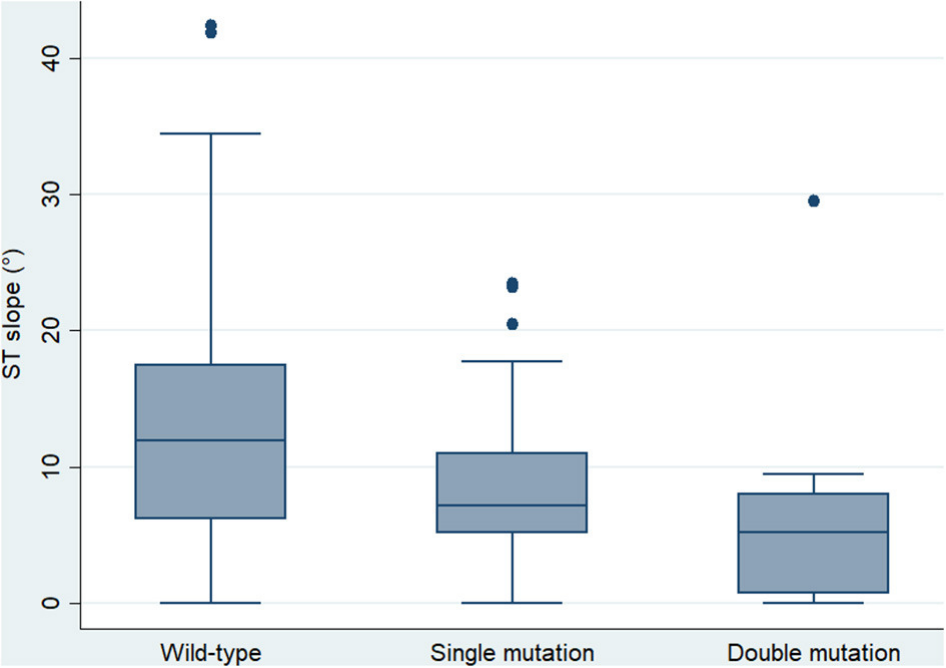
Box plot showing the median and interquartile range of the ST slope in wild-type controls and single- and double-mutation carriers (*p* = 0.001).

Digital electrocardiograms were obtained from carriers of 15 different mutations (5 pathogenic, 5 VUS, 5 benign or likely benign, Table 1). Among 41 genotype-positive/phenotype-negative individuals, 12 bore 2 different mutations in desmosomal genes. Their electrocardiographic parameters were compared with those of single-mutation carriers and the wild-type relatives (Table 3). The averaged ST slope showed a significant difference between the three groups: 7.15° (5.14–11.05), 5.18° (0.73–8.01), and 11.46° (3.94–17.49) (single mutation, double mutation, and wild type, respectively), *p* = 0.005 (Figure 1).

**Table 3:** Quantitative analysis of the main electrocardiographic parameters in single- and double-mutation carriers and wild-type relatives.

	**Simple carriers (*N* = 29)**	**Double carriers (*N* = 12)**	**Wild type (*N* = 73)**	***p***
Sex (male)	14 (48.27%)	8 (66.67%)	45 (61.64%)	0.5590
Age (years)	36 (28–48)	35 (25–56)	34 (26–47)	0.8080
BMI (kg/m^2^)	24.01 (21.48–25.50)	23.67 (22.66–26.40)	23.41 (21.80–26.85)	0.7919
**ECG**				
IRBBB	14 (48.27%)	0 (0%)	23 (31.51%)	0.012
CRBBB	0 (0%)	0 (0%)	3 (4.11%)	0.0382
QRS (ms)	84 (78–94)	86 (76–100)	84 (80–84)	0.9288
QRS V1–3 (S-offset) (ms)	38 (36–44)	38 (28–42)	38 (34–42)	0.5667
QRS fragmentation (*n*)	1 (0–2)	0	1 (0–1)	0.9579
ST slope (°)	7.15 (5.14–11.05)	5.18 (0.73–8.01)	11.46 (3.94–17.49)	0.0054
TWI (*n*)	1 (1,2)	1 (0–2.5)	1 (1,2)	0.8822
T-wave amplitude (mV)	0.223 (0.119–0.277)	0.171 (0.11–0.30)	0.214 (0.139–0.285)	0.7696
T-wave area (μVs)	18.91 (12.30–29.18)	11.03 (6.37–33.44)	22.35 (12.77–32.98)	0.4965
T-wave dispersion (μVs)	2.36 (1.13–4.96)	2.96 (2.41–10.38)	1.82 (0.81–4.79)	0.1936

*BMI, body mass index; IRBBB, incomplete right bundle branch block; CRBBB, complete right bundle branch block; TWI, T-wave inversion*.

The ST slope significantly correlated with the T-wave voltage in mutation carriers (*r* = 0.376, *p* = 0.021) but not in the wild-type group (*r* = 0.074, *p* = 0.570). Other electrocardiographic variables did not demonstrate any correlation with the ST slope.

**Figure 2:**
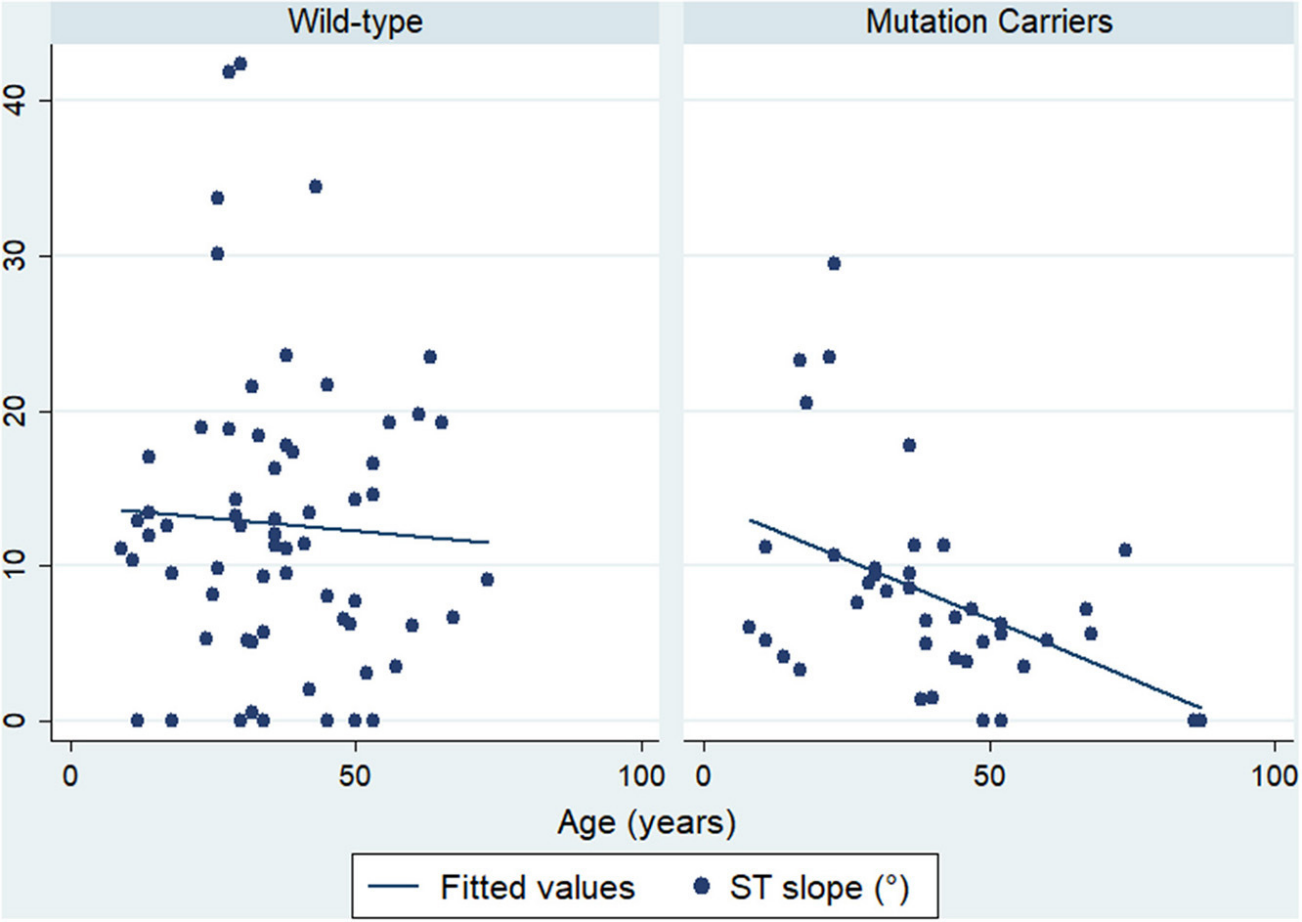
Correlation between ST slope and age. Mutation carriers are presented on the right side and wild-type on the left side.

Of note, a negative correlation between the ST slope and age was present in mutation carriers (*r* = −0.353, *p* = 0.022) but not in wild-type individuals (*r* = −0.053, *p* = 0.672) (Figure 2).

The effect of the genetic mutations on the ST slope was estimated with a linear regression model. Potential confounding variables such as age, sex, body mass index, and the type of mutation (classified into truncating or no truncating mutations) were included in the model. The presence of double mutations was independently associated with a flatter ST slope (b = −7.86, *p* = 0.002), while this effect was not significant in the case of single mutation carriers (b = −2.344, *p* = 0.206) (Table 4).

**Table 4:** Linear regression models.

	**Model 1**	**Model 2**
	**ST slope**	**ST slope**
**Genetic status**		
0. Control	0	0
1. Simple mutation	−2.344	−1.904
	(1.839)	(1.955)
2. Double mutation	−7.862**	−7.641**
	(2.416)	(2.678)
**Sex**		
0. Female	0	0
1. Male	3.781*	3.516*
	(1.614)	(1.737)
**BMI (kg/m**^**2**^**)**	0.161	0.115
	(0.261)	(0.271)
**Age (years)**	−0.0705	−0.0701
	(0.0587)	(0.0638)
**Type of mutation**		
0. Non-truncating		0
1. Truncating		−0.843
		(1.872)
Constant	8.730	10.06
	(5.373)	(5.600)
*N*	84	75
*R*^2^	0.194	0.180

*The included variables were ST slope (dependent variable), genetic status (independent variable) and sex, body mass index, and age (as potential confounders). A second model (on the right) added the type of mutation (truncating/non-truncating). Non-carriers and female sex are considered the reference for the variables sex and type of mutation, respectively. Standard errors are shown in parentheses.*

* *p < 0.05,*

** *p < 0.01*.

## Discussion

The diagnosis of arrhythmogenic cardiomyopathy poses a challenge in the clinical practice due to a wide phenotypic spectrum that encompasses right-sided (1) and left-sided or biventricular forms (9, 10). Despite current efforts to stratify the risk of sudden death in these patients, it is well-recognized that affected individuals may die suddenly at an early phase of the disease when electrocardiographic and structural abnormalities are usually absent.

Therefore, the identification of the mutation carriers who will develop the disease is challenging. Magnetic resonance imaging (11) and speckle tracking (12) have shown minor abnormalities in healthy mutation carriers, although they are not routinely used in the family screening due to the lack of reproducibility of the speckle tracking and the cost and risk of overdiagnosis of non-pathological findings such as subtle wall motion abnormalities in cardiac magnetic resonance.

Once the diagnosis of arrhythmogenic cardiomyopathy is established, carrying out a genetic study, as well as a complete cascade family screening, is recommended to identify relatives at risk. This strategy also helps make a definitive diagnosis in probands with a thus far borderline phenotype (7).

As electrocardiographic abnormalities usually precede the development of overt structural cardiomyopathy, we aimed to identify signs of the electric “concealed stage” of the cardiomyopathy through a detailed analysis of digital electrocardiograms. Despite the presence of complete right bundle branch block, negative anterior T-waves, terminal activation delay > 55 ms in V1–V3 being frequently found in arrhythmogenic cardiomyopathy patients (13, 14), data on the prevalence of these electrocardiographic abnormalities in genotype-positive/phenotype-negative individuals are lacking. Furthermore, studies that compare mutation carriers with non-mutation carriers from the same family are pertinent to understand the phenotypic effect of mutations and variants.

We retrospectively evaluated the digital electrocardiograms of carriers of pathogenic mutations that did not show either evidence of structural disease on imaging or electrocardiographic abnormalities included in the current diagnostic criteria. Accurate QRS, ST, and T-wave measurements were obtained through digital electrocardiographic analysis thereby allowing a more sensitive approach to that provided by the analysis of conventionally recorded electrocardiograms. QRS terminal activation delay, averaged ST slope in the 12 leads, and T-wave voltage were therefore quantitatively assessed, which should overcome the subjectivity in the interpretation of subtle electrocardiographic abnormalities.

The initial hypothesis of the study was that of healthy genotype-positive/phenotype-negative individuals showing a longer QRS duration and terminal activation delay, a flatter ST slope, and smaller T-wave amplitude than wild-type relatives. We hypothesize that mutations and variants associated with the development of arrhythmogenic cardiomyopathy may play a role in early GAP junctions and connexin-43 remodeling (15) as well as intracellular ion mishandling (16), which may precede the development of electrocardiographic abnormalities (right bundle branch block and T-wave inversion) and further gross structural abnormalities as the clinical phenotype progresses. A proportion of the carriers included in the study bore non-pathogenic variants, which may either act as modulators of the phenotypic expression of the disease or cause subtle electrocardiographic abnormalities.

**Figure 3:**
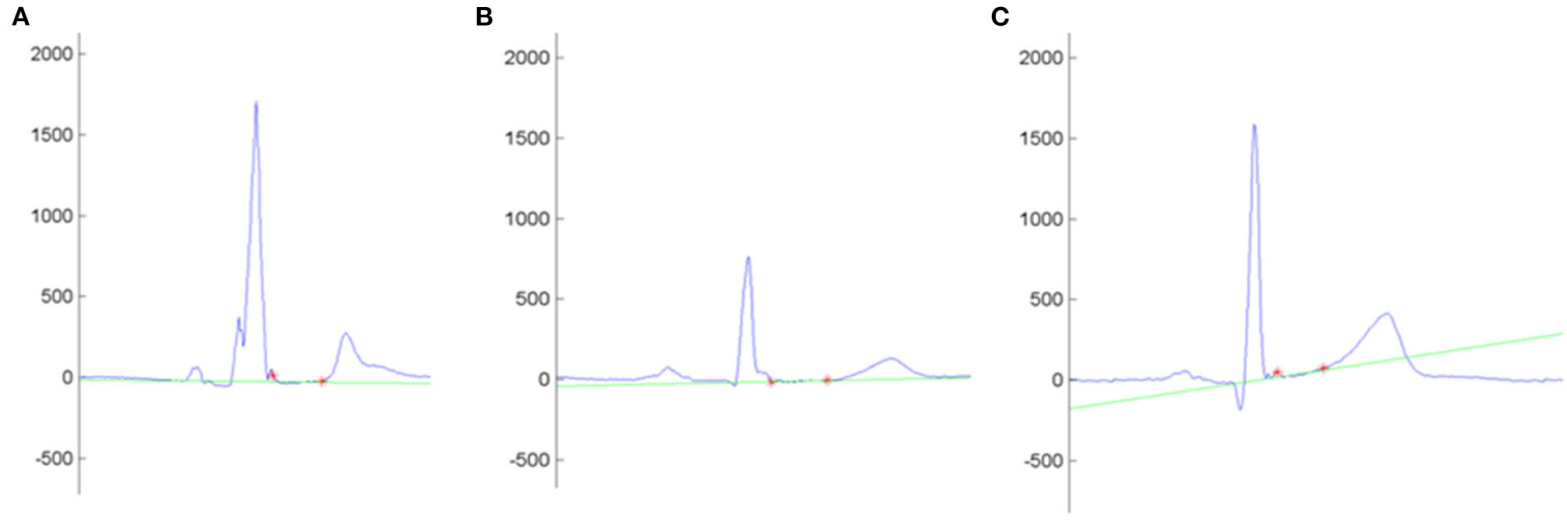
Example of the automatic measurement of the ST slope in the lead I. Y-Axis shows the voltage (mV). Red asterisks mark the QRS offset and the T-wave onset. The intersection line (green) determines the ST-slope angle with the isoelectric line. **(A–C)** shows the ST slope of an arrhythmogenic cardiomyopathy patient (−0.4°), a non-affected mutation carrier (1.9°), and a control (9.3°), respectively.

Our study demonstrated the trend of genotype-positive/phenotype-negative relatives to show a flatter ST slope, more pronounced in the case of double-mutation carriers. The effect of a double-hit mutation on the ST slope remained significant after correction for potential confounders such as age, sex, and body mass index. Interestingly, we did not find differences between mutation carriers and wild-type individuals in any of the parameters included in the current of former diagnostic criteria, the number of QRS fragmentations, or the T-wave amplitude. In view of these findings, we propose that ST-slope flattening is an early electrical abnormality in relatives with a significant genetic burden (Figure 3). Gene carriers bearing this repolarization pattern may benefit from a thorough cardiological study [including cardiac magnetic resonance, Holter monitoring and exercise test (17)] and a more frequent follow-up than other healthy relatives.

The analysis of the ST-segment morphology has proved to be a useful tool in the differential diagnosis between arrhythmogenic cardiomyopathy and the physiological adaptation to competitive sport commonly known as athlete's heart. Of note, a convex upward morphology (positive ST slope) in anterior leads suggests a physiological remodeling, whereas a flat or down-sloping ST (negative ST slope) suggests the diagnosis of arrhythmogenic cardiomyopathy (18). Repolarization abnormalities in arrhythmogenic cardiomyopathy patients classically involves right precordial leads. However, as biventricular or left-sided phenotypes may also affect inferior and left precordial leads, the average ST slope of the 12 leads was included in the analysis.

In order to evaluate the ST slope beyond the dichotomic classification between positive or flat/negative slope, we support a quantitative approach rendered by the processing of digital electrocardiograms, which eliminates the interobserver bias.

As arrhythmogenic cardiomyopathy typically shows an incomplete penetrance, it would be reasonable to expect a progressive flattening of the ST slope during follow-up, which is beyond the scope of this research. Whether ST flattening precedes a future T-wave inversion will require further investigations. In this regard, we observed a negative correlation between age and ST slope in mutation carriers, which supports that hypothesis.

This research has several limitations. First, this is a non-matched case–control study that includes a small number of individuals. Second, it was not possible to identify the mutation carriers who will develop further electrical or structural abnormalities on the basis of ST. This hypothesis will require further investigations. Third, the interpretation of the genetic analysis is challenging in arrhythmogenic cardiomyopathy due to a significant prevalence of desmosomal variants in the general population. Some of the variants included are not disease causing by themselves although may contribute to the phenotype of the disease.

In conclusion, we proposed that a detailed and thorough analysis of digital electrocardiograms in genotype-positive/phenotype-negative individuals renders the identification of early electrocardiographic abnormalities, which may portend an early phase of the disease. ST-slope flattening is the earliest abnormality found in double-hit mutation carriers.

## Data Availability Statement

The raw data supporting the conclusions of this article will be made available by the authors, without undue reservation.

## Ethics Statement

The studies involving human participants were reviewed and approved by Virgen de la Arrixaca Ethics Committee. The patients/participants provided their written informed consent to participate in this study.

## Author Contributions

JL-A: conceptualization, methodology, writing-original draft, and formal analysis. DL-C: resources and conceptualization. MM: methodology and genetic testing. JaG-B: conceptualization, software, methodology, and formal analysis. JuG-B: conceptualization, data curation, and writing—original draft, resources. All authors contributed to the article and approved the submitted version.

## Conflict of Interest

The authors declare that the research was conducted in the absence of any commercial or financial relationships that could be construed as a potential conflict of interest.
